# Ferromagnetic order controlled by the magnetic interface of LaNiO_3_/La_2/3_Ca_1/3_MnO_3_ superlattices

**DOI:** 10.1038/s41598-023-30814-6

**Published:** 2023-03-08

**Authors:** S. Soltan, S. Macke, S. E. Ilse, T. Pennycook, Z. L. Zhang, G. Christiani, E. Benckiser, G. Schütz, E. Goering

**Affiliations:** 1 Physics Department, Faculty of Science, Helwan University, Helwan, Cairo, 11798 Egypt; 2 Max Planck Institute for Intelligent Systems, Heisenbergstr. 3, 70569 Stuttgart, Germany; 3 Max Planck Institute for Solid State Research, Heisenbergstr. 1, 70569 Stuttgart, Germany; 4 EMAT, University of Antwerp Campus Groenenborger, 2020 Antwerp, Belgium; 5 Faculty of Physics, University of Vienna, Boltzmanngasse 5, 1090 Vienna, Austria; 6 Erich-Schmid-Institute of Materials Science, Austrian Academy of Sciences, Jahnstraße 12, 8700 Leoben, Austria

**Keywords:** Materials science, Nanoscience and technology, Physics

## Abstract

Interface engineering in complex oxide superlattices is a growing field, enabling manipulation of the exceptional properties of these materials, and also providing access to new phases and emergent physical phenomena. Here we demonstrate how interfacial interactions can induce a complex charge and spin structure in a bulk paramagnetic material. We investigate a superlattice (SLs) consisting of paramagnetic LaNiO_3_ (LNO) and highly spin-polarized ferromagnetic La_2/3_Ca_1/3_MnO_3_ (LCMO), grown on SrTiO_3_ (001) substrate. We observed emerging magnetism in LNO through an exchange bias mechanism at the interfaces in X-ray resonant magnetic reflectivity. We find non-symmetric interface induced magnetization profiles in LNO and LCMO which we relate to a periodic complex charge and spin superstructure. High resolution scanning transmission electron microscopy images reveal that the upper and lower interfaces exhibit no significant structural variations. The different long range magnetic order emerging in LNO layers demonstrates the enormous potential of interfacial reconstruction as a tool for tailored electronic properties.

## Introduction

The interest in interfaces between half-metal ferromagnet manganites La_2/3_Ca_1/3_MnO_3_ (LCMO) and the paramagnetic metal LaNiO_3_ (LNO) is stimulated by fundamental questions aiming to understand the relation between the magnetic, electronic, and crystallographic structures^1–5^. This is of special interest to study to understand the mechanisms of magnetic interface structures, electronic- and spin-transport across the FM/non-FM barrier^6–11^ including for potential applications of hybrid oxide ferromagnet/non-ferromagnet (FM/non-FM) structures in spintronic devices.

For powder LNO samples only paramagnetism has been found which is therefore the only member of the perovskite nickelates family lacking any magnetic order in its bulk form^12^. In contrast, in LNO single crystals the presence of an antiferromagnetic ground state has been reported^13^, but these results are controversial and debated in terms of oxygen stoichiometry^14^. The magnetic reconstruction at the interface between a ferromagnetic and a non-ferromagnetic oxide enrich the physics of spin transport, i.e. spintronic devices^15–19^. At such interfaces net ferromagnetic moments can appear in otherwise non-ferromagnetic ordered layers. Often the exchange-bias plays an important role in such induced magnetism heterointerfaces^19,20^, resulting from an exchange anisotropy present at the interface between two materials with competing magnetic interactions^1–5^. Typically, it is associated with the interfacial coupling between a ferromagnet and an antiferromagnet when field-cooled through the Néel temperature. Other biasing effects can also appear at other interfaces (for example, owing to the antiferromagnetic coupling between two ferromagnets) or even in materials with inhomogeneous magnetic phases^21–25^. Beyond an exotic spin transport response, the presence of magnetic moments in the barrier material can also influence magnetic switching, which produces a complex nano-magnetic state at the interface. The reconstructed chemical bonds have been proposed to give rise to an induced magnetic state at the non-magnetic barrier^26^. So far only ¼ ¼ ¼ antiferromagnetic order has been reported on LNO-RXO superlattices with 2 u.c. of LNO^27–31^.

Here we report XRMR measurements at the Mn L_2,3_ and the Ni L_2,3_ edges in order to provide detailed knowledge about the magnetic interface structures of pesudocubic (001) oriented layers of paramagnetic LNO and ferromagnetic LCMO. We deduced the distribution of the Mn magnetic moments and observed a clear oscillating magnetic Ni moment induced at the LCMO/LNO interface. Element specific XRMR hysteresis loops confirmed the coupling of the Mn–Ni moments, which is also confirmed by the observation of exchange bias effects at low temperatures. These results bring key insights into the dependence of magnetic moments in the superlattice as a function of magnetic field, and temperature, and suggest routes for the optimal combination of ferromagnetic and barrier effects in future spin transport devices. So far, magnetic order (AFM) could be induced for critical LNO thickness above 2-unit cells (u.c.) by strain for PNO-PAO Sls as a consequence of reduced dimensionality^1–3,27–29^.

**Figure 1:**
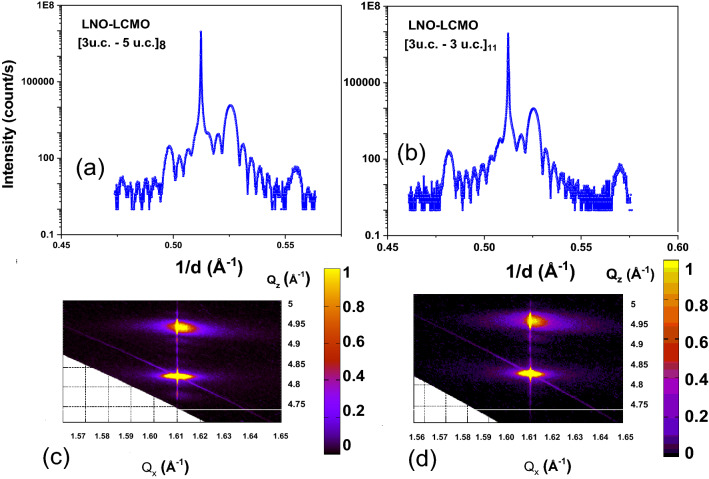
(**a**,**b**) shows the high-resolution hard X-ray structural characterization for SLs consisting of [3-LNO–5-LCMO]_8_ and [3-LNO–3-LCMO]_11_. (**c**,**d**) shows the reciprocal space mapping (RSM) around (013)-STO, for both superlattices (**a**) and (**b**) respectively.

The superlattices were grown on a single crystalline SrTiO_3_ substrate using pulsed laser deposition. Systems consisting of eight repetitions of 3 u.c. of LNO and 5 u.c. of LCMO [3-LNO–5-LCMO]_8_ and 3 u.c. of LNO and 3 u.c. of LCMO [3-LNO–3-LCMO]_11_ were studied. Figure 1a and b shows the high-resolution hard X-ray structural characterization for the SLs. The analysis of the X-ray data of Fig. 1a and b confirm that the c-axis lattice constant of LCMO and LNO is calculated as 3.832 Å and 3.830 Å, respectively, while the bulk values are 3.857 Å-LCMO and 3.850 Å-LNO. The results indicate that both LCMO and LNO are under tensile/compressive strain. In order to distinguish between both states, strain mapping is also shown in Fig. 1c and d by reciprocal space mapping (RSM) around (013) Bragg reflections of the STO substrate. RSM has been taken to quantify the strain state in the superlattices by using a four-circle X-ray diffractometer using a rotating Cu anode source running at a cathode voltage of 45 kV and filament current of 120 mA. In Fig. 1c and d the intense peak around Q_x_ = 1.61 Å^−1^ and Q_z_ = 4.83 Å^−1^ corresponding to the (013) Bragg reflection of the STO substrate, while the peak at Q_x_ = 1.61 Å^−1^ and Q_z_ = 4.95 Å^−1^ originates from the combined structure of LCMO–LNO layers due to the overlap between the reflections of the two layers. In this case, the in-plane lattice parameters of LCMO and LNO match perfectly those of STO, and the superlattice is strained. The highest intensity at Q_x_ of the substrate is due to interference with the truncation rod, but the superlattice peak center is at slightly higher Q_x_ values, as shown in Fig. 1c and d.

**Figure 2:**
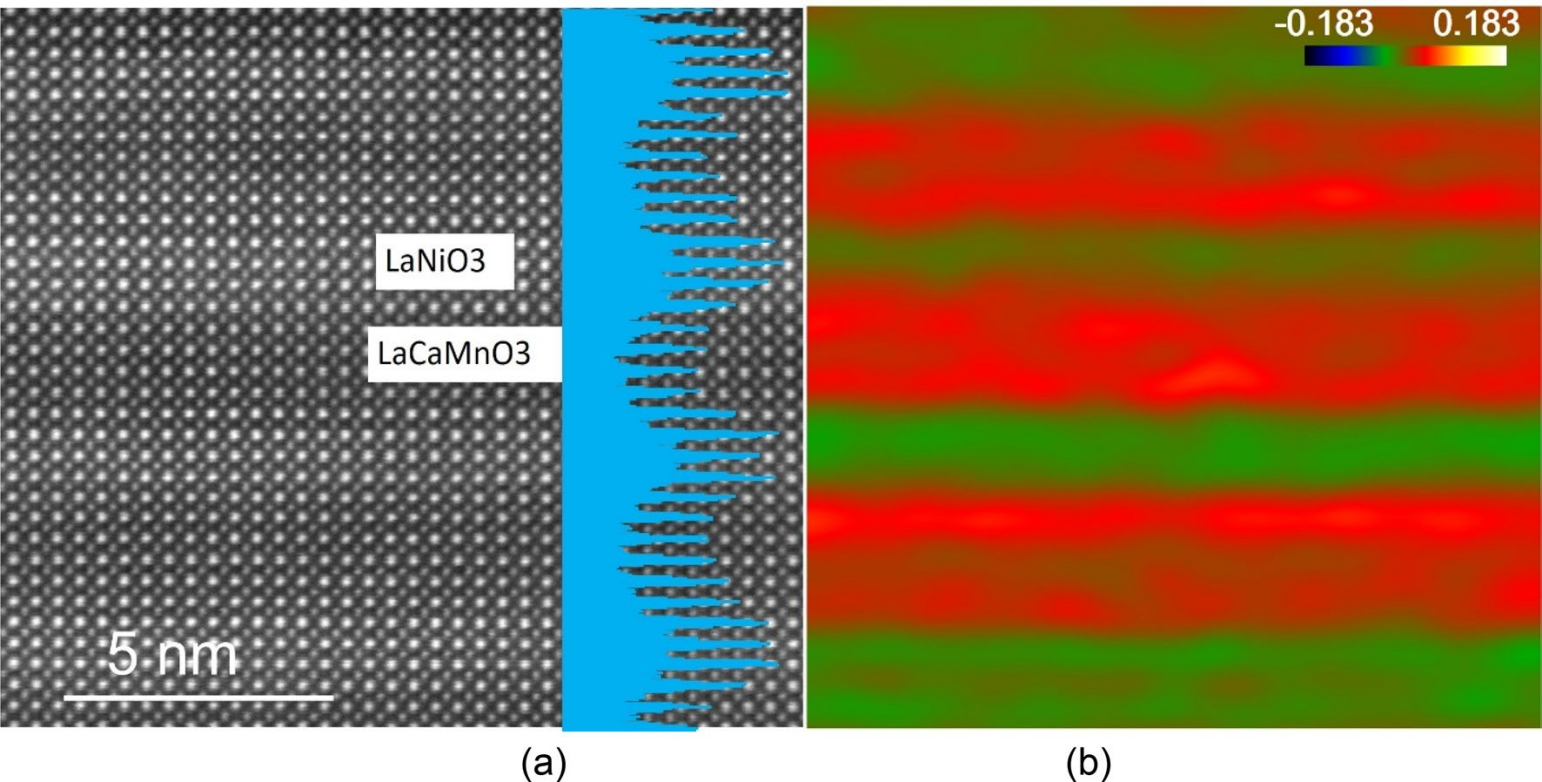
(**a**) High resolution HAADF STEM with an overlaid intensity profile shown in blue. (**b**) The strain map along the film growth direction.

High-angle annular dark-field (HAADF)—scanning transmission electron microscope (STEM) imaging is presented in Fig. 2a. The LNO layers can be identified by their brighter overall contrast compared to the LCMO layers (as labelled) due to the lower La concentration in the LCMO as the HAADF intensity increases as approximately the square of the atomic number. The overlaid intensity profile acquired from the horizontal integral of the HAADF image shows the structure modulation of LNO and LCMO layers on the substrate. Spectrum imaging with electron energy loss spectroscopy is shown in Fig. S1. It is clearly seen that interfaces gradually change, are generally symmetric and do not show abrupt changes in the interface structure. This suggests a certain amount of Ni and Mn intermixing occurred within one unit cell across the interface. The intensity also indicates the layer thicknesses, i.e. that we indeed have 3 u.c. of LaNiO_3_ and 5 u.c. La_0.67_Ca_0.33_MnO_3_ and this is repeated in almost 8 full periods on the SrTiO_3_ substrate. A strain map along the film growth direction (e_yy_) is shown in Fig. 2b derived from the HAADF image showing that LNO is compressed (green) while LCMO is expansively strained (red) along the growth direction. We also note some variation of the strain within the individual layers.

**Figure 3:**
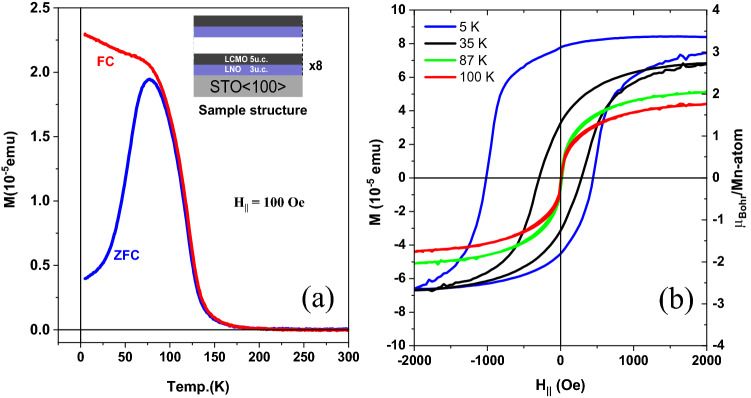
(**a**) Magnetization versus temperature for the [3 u.c. LNO–5 u.c. LCMO]8 superlattices, with the in-set illustrating the sample structure. (**b**) Magnetization versus magnetic field (left-axis) and relative Bohr-magneton (µB/Mn-atom) (right-axis) for the same sample at 5 K, 35 K, 87 K, and 100 K.

Figure 3a shows the zero-field-cooling (ZFC) and field-cooling (FC) magnetization versus temperature curves, with an in-plane oriented external field of H_//-plane_ = 100 Oe for the superlattices. It shows a reduced superlattice Curie temperature T_c-SL_ ~ 175 K compared to the LCMO bulk value of T_c-bulk_ = 275 K.

Figure 3b shows (left-axis) the magnetization versus magnetic field and also the Bohr-magneton (µ_B_/Mn-atom) (right-axis) derived by normalizing the total magnetization as described in more details below. Furthermore, the transport and magneto-resistance of the same superlattices structure [3 u.c. LNO–5 u.c. LCMO]_8_ shows a metallic behavior in all temperature range (see supplement part Fig. S8).

**Figure 4:**
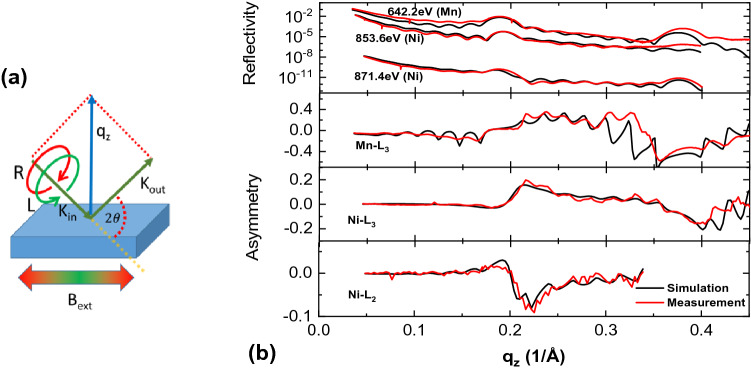
(**a**) Experimental θ–2θ geometry, showing the incoming and outgoing photon K vector of circular polarized light, where the magnetization has been flipped in plane. q_Z_ is the vector for the momentum transfer. (**b**) Fitted reflectivity and asymmetry curves the normed asymmetry is the difference between for reflectivity curves measured for left and right circular light on the Mn-edge.

Next, we determine the depth profiles from X-ray reflectivity and XRMR scans, which were performed at the Mn-L_3_ and Ni-L_2,3_ resonances as well as in off-resonant energy regions. The sketch of the scattering geometry and the results for the reflected intensity as a function of the momentum transfer vector q_z_ are shown in Fig. 4.

The reflectivity curves were modeled by an atomic density model using appropriate non-magnetic and magnetic scattering factors, respectively. Hereby, the complex refractive indices of LNO, LCMO, and STO were created by using tabulated off-resonant calculated scattering factors^28,29^. The spectral shape of the Mn resonance was determined from X-ray absorption spectroscopy (XAS) signals, fitted and merged to tabulated off-resonant values of the index of refraction. This well-known procedure provides the absorptive scattering part. The corresponding dispersive scattering factors of Mn were then obtained by Kramers–Kronig transformation as described elsewhere^31^.

Due to the spectral overlap and self-absorption phenomena of the La-M_4,5_ and the Ni-L_2,3_ edges a straightforward extraction of reasonable Ni and La spectra from the XAS signal was simply not possible. The La^3+^ M_4,5_ spectrum is quite simple and independent from the chemical surrounding. Hence, the scattering factors of La have been retrieved from a pure LaAlO_3_ substrate. On the other hand, Ni scattering factors were taken from a PrNiO_3_ film in where Ni has the same valence state as in LNO^30^. For all other elements tabulated off-resonant values were used.

The chemical depth profile was determined by fitting only one parameter set of thicknesses for each LNO and LCMO layer, and the corresponding roughness at each interface, at the STO interface, and at the vacuum surface. Together, with one general scaling factor, which considers slight continuous roughness variations from the substrate to the vacuum, we used only 7 fitting parameters and scanned the whole parameter space by applying a generic fit algorithm and the sum of squared residuals as error-bars.

**Figure 5:**
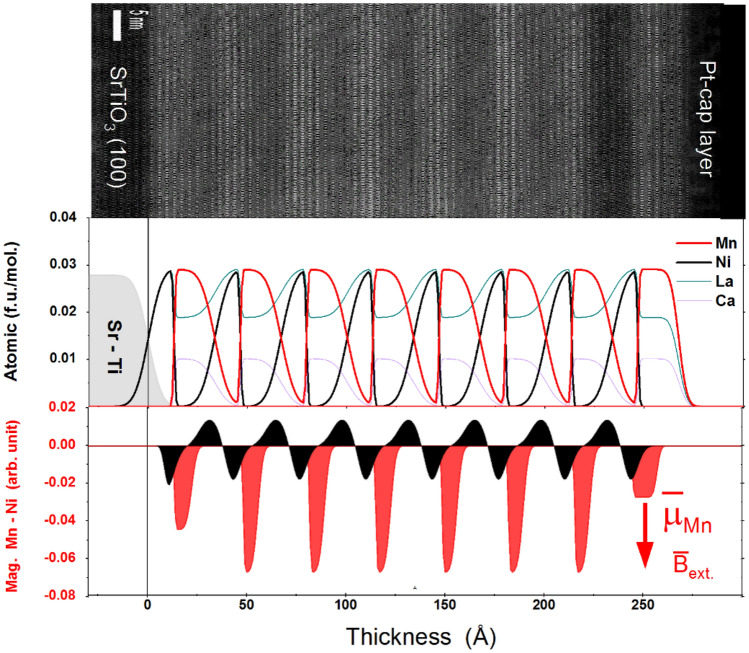
The upper part shows the corresponding HAADF and middle panel the chemical profiles for all present elements in the fitted [3 u.c. LNO–5 u.c. LCMO]8 superlattice. The model is assumed to be periodic and reproducible for the eight repetitions (see text). The stoichiometry is fixed for bulk STO, LCMO and LNO, but roughness and thickness are variable. The lower part shows the corresponding Mn magnetic profile at T = 35 K, Where the red arrow indicates the relative orientation between the magnetic field and the Mn magnetization.

Figure 5 shows the corresponding measurements and the best fits, performed using ReMagX^30–32^. The corresponding HAADF and chemical profiles are shown in the upper and middle panel of Fig. 5 respectively. The fit confirms the expected 5/3 partition with the thicknesses of 20.5 Å ± 1 Å and 12.9 Å ± 1 Å for LCMO and LNO respectively.

Interestingly, the obtained XRMR roughness for LNO/LCMO and the LCMO/LNO interfaces is significantly different. The XRMR based wide and narrow interfaces also differ to the STEM results shown in Fig. 2, where both interfaces are much more similar. If we only fit the off resonant XRMR curves the fit quality is less sensitive to the interface width and more symmetric interface profiles do provide almost similar but still fewer good results. Especially the circular polarized resonant fits suggest these large differences in the chemical interface mixing, as we will denote in the following as wide and narrow. As these resonant XRMR curves are most sensitive to the local electronic structure, they are a strong indication that a spatial variation in the optical properties due to valence variations could be responsible for the observed strong differences. Or in other words, a “wide interface” should reveal a wide variation of the optical properties, while a “narrow” interface should have an abrupt change. Actually, an asymmetric interface reconstruction has been already observed for this kind of superlattices^33^. The sharp interface has a roughness of 0.65 Å ± 0.4 Å, while the wide has 5.6 Å ± 3 Å.

Of course, further assumptions like contaminations and different thicknesses and for each double layer would further improve the fit, but are questionable as these parameters are not conclusively confirmed by observable features in the curves. For the sake of clarity and plausibility we stay here with the already explained seven parameters fit.

To determine the magneto-optical depth profile an electromagnet producing a homogenous magnetic field of 0.1 T has been used to align the magnetization in the film plane along the measurement scattering plane, as indicated in Fig. 4a. The change of the soft X-ray resonant optical properties due to the XMCD effect, two different reflectivity curves at the Mn-L_3_ resonance were measured by using left *R*_*l*_ and right circular *R*_*r*_ polarized light. The lower right part of Fig. 4b shows the related Mn magnetic asymmetry defined as $A=\frac{Rl-Rr}{Rl+Rr}$ and the corresponding fit.

During the magnetic fitting, the magnetic depth profile was assumed to have the same overall periodicity as the superlattice. Each periodic LCMO/LNO block was assumed to have only one magnetic continuum layer with in-plane magnetization and free thickness, position and magnetic roughness. As model inputs for Mn, the magneto optical constants were determined by the corresponding XMCD spectra, similar to the above described method for the non-magnetic optical constants (see Supplemental Fig. S7). Further details on this procedure have been described elsewhere^31^.

The magnetic fit profiles are shown in the lower part of Fig. 5, revealing a homogeneous magnetization in the stoichiometric region of the LCMO-layer stacks (right part at about 250 Å) and a sharp (0.56 Å ± 0.3 Å) and rough (2.0 Å ± 1 Å) interface, consistent with the chemical profile. In addition, the Mn magnetization is also reduced at the rough side, giving something similar than a magnetic dead layer. We also want to mention that this might be related to a local AFM Mn order, which we could not distinguish by XRMR. Note, that the fit boundaries were completely free also allowing for physically implausible profiles. This further strengthens the correctness of the thickness and roughness of the chemical profile.

The right part of Fig. 4 shows the magnetic Ni L_2,3_-edge asymmetries measured and the corresponding asymmetry fits. In case of Ni we use three layers with different magnetization in each LNO slab, as is necessary to provide the antiparallel layer magnetizations, together with the relatively flat region in the Ni layer center. The Ni magnetization profile have been determined completely independently for the Ni-L_3_ and Ni-L_2_ edges by using generic algorithms. These reveal that in the Ni film at least two layers with different magnetization direction occur. At the wider interface the Ni tends to have a magnetization with antiparallel orientation with respect to the external field or the Mn magnetization, whereas at the sharp interface the magnetization is aligned parallel to the field (Mn magnetization). The data is also consistent with the observed very weak Ni XMCD signal as the oppositely aligned magnetic Ni layers almost cancel the corresponding Ni XMCD effect (see Supplemental Fig. S7). Both best fits revealed almost the same magnetic Ni profile, which are for both edges in almost perfect agreement with the experiment. As both independently fitted edges reveal the same consistent profile, it is a good model. For further support, we also tried to fit the Mn and Ni data with symmetric magnetization profiles. These test results provided inconsistent profiles, which are clearly distinct for both edges and reduced fit quality. Further information is provided in the Supplemental sections 3 and 4.

Now we want to normalize the SQUID results by the magnetic and nonmagnetic Mn profiles shown above be XRMR. The total number of Mn ions has been determined by the integral of the nonmagnetic Mn profile, which is scaled to the nominal Mn concentration of 0.029 mol/cm^3^. By multiplying this integral by the sample surface area of 0.5 cm x 0.5 cm we get 1.2 × 10^–8^ mol of Mn ions. As the XRMR has been measured at 35 K we calculate the SQUID saturation magnetic moment Mn (35 K) = 1.02 µ_B_. On the other hand, from the magnetic Mn XRMR profile, we clearly see that Mn is not saturated on the broader site of the Mn/Ni interfaces and especially at the upper and lower interfaces. Now we normalize the magnetic XRMR Mn profile at the peak intensities also to the nominal Mn concentration of 0.029 mol/cm^3^, assuming that the highest value in the Mn magnetization profile is related to full saturation. This gives us an effective number of fully magnetized Mn ions of 4.05 × 10^−9^ mol. If we rescale the Mn magnetic moment (35 K) by this ratio, we get 1.02 µ_B_*1.2/0.4 = 3.0 µ_B_/Mn ion. For the 5 K SQUID magnetization curve in Fig. 3 we clearly see a shift of the magnetization to positive magnetization values. Therefore, we estimate the saturation from the left and right-side average at 200 mT giving 7.5 × 10^−5^ emu. This gives us the 5 K saturation magnetization of 3.0 µ_B_/Mn * 7.5 × 10^−5^ emu (at 5 K)/6.75 × 10^−5^ emu (35 K) = 3.54 µ_B_/Mn. This is within the estimated error bar of 0.3 µ_B_/Mn in perfect agreement with reported bulk values of 3.6 µ_B_/Mn, see Bibes et al.^34^ and also consistent to the high spin valence states at the narrow interface state discussed below. This consistency also demonstrated the reliability of the obtained Mn XRMR profiles (magnetic and nonmagnetic). We also want to emphasize that the strong magnetization reduction at the rough interface might be related to Mn AFM order, which would be an additional explanation for the observation of exchange bias at low temperatures. Nevertheless, also the antiparallel Ni exchange interaction might be responsible for reduced FM order at this side. The details related to the exchange-bias are shown in the supplementary FORC part Fig. S6.

## Discussion

The main result is the observation, of two oppositely magnetized Ni interface layers that correlate with two different charge or valence roughness’s. It was clearly identified, that the Ni magnetic moment is aligned parallel to the Mn moment at the sharp interface, and antiparallel at the wide interface. On the other hand, the magnetization of Mn is strongest at the sharp interface and in contrast almost vanished at the wide interface. The Mn magnetization on the surface of the sample (at z = 25 nm) is reduced and more rectangular in shape. The Ni XRMR signal is much weaker indicating two antiparallel configurations of almost the same strength with the consequence that Ni does not contribute significantly to the total magnetization. In order to verify this, we compare the integrated Mn magnetization profile, which should represent the total Mn magnetization and the total sample magnetization. Therefore, we just integrate the Mn signal of the multilayer structure and set this value to the total magnetization of 1.4 µ_B_ measured by SQUID at the temperature of 35 K. With this procedure we are able to translate the arbitrary scale in Fig. 5 directly to the Mn atomic moments as shown on the bottom panel.

**Figure 6:**
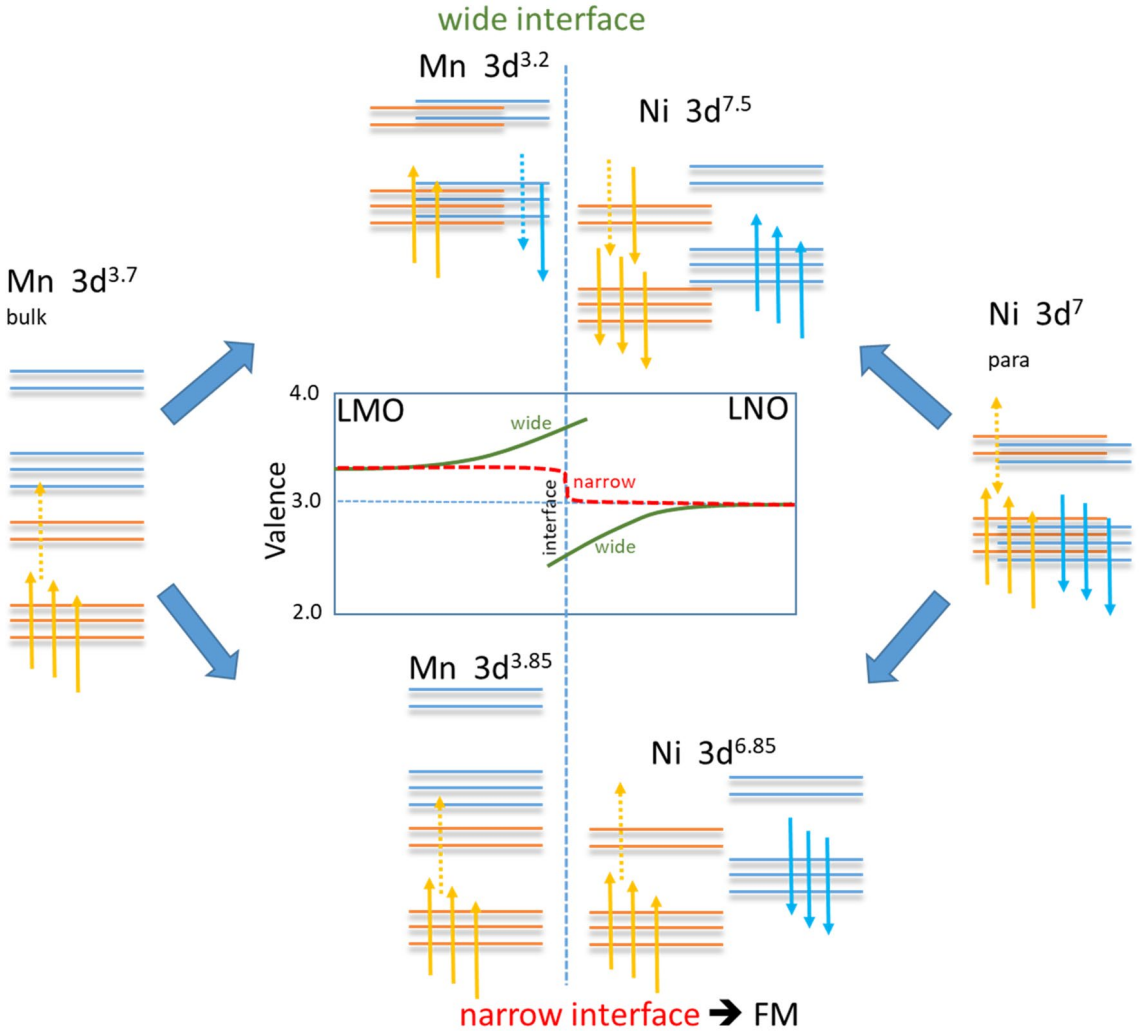
On the left and the right the simplified electronic structure of bulk Mn in LCMO and Ni in LNO are shown. The center part shows the electronic structure of both in contact at the wide and the narrow interface. Dotted arrows represent the configurational averaged non-integer valence, where the 1/3 doping is presented simplified by 3d^3.7^ and not as 3d^3.667^. The center graph shows schematically the valence as a function of distance to the interface for the as wide and narrow classified interfaces. For the wide interface valence distributions are extracted from Ref.^2^.

In order to understand this complex behavior a simplified electronic structure model is presented in Fig. 6. On the left (right) the electronic structure of bulk Mn high spin (Ni paramagnetic) is presented, respectively. The bulk values are related to the 1/3 Ca doping and the undoped LNO. As it has been reported in Ref.^2^ a continuous and about 4 nm wide range charge variation has been observed, where the Mn increases its valence up to Mn^3.8+^ (3d^3.2^) by losing about 0.5 electrons, while Ni decreases its valence by the same amount from Ni^3+^ (3d^7^) to Ni^2.5+^ (3d^7.5^). This is in very good agreement with our wide interface. As it is well known from the LCMO phase diagram, Mn with more than 0.5 of Ca doping switches to AFM order, we would expect to have no FM present anymore at the Mn site by this kind of charge variation. Indeed, the Mn magnetization profile suggests the absence of FM order at the wide interface. Here at the wide interface condition we also found AFM order between the average Mn magnetization and the Ni magnetization at the wide interface in contrast to Ref.^2^.

On the other hand, at an electronically sharp interface, in terms of valence state, one would expect to have almost the same valence present up to the interface, and at the interface itself one can find an average valence for both parts, as presented in the lower central part of Fig. 6. This means Mn will get 0.15 electrons from Ni changing Mn to 3d^3.85^ (Mn^3.15+^) and Ni to 3d^6.85^ (Ni^3.15+^). For Mn the relevant effective doping is still in the high exchange split FM region of the LCMO phase diagram, one would expect a FM ordered Mn state with an increased Mn moment, because its electronic configuration is closer to half filling. Interestingly, both Ni states are in a doped region, which can provide double exchange like FM interactions. As in the narrow interface both sides have non-fully occupied states, Ni is able to provide FM order. In the case of the Mn high spin configuration, Mn will provide exchange coupling to Ni with a full magnetic moment related to the uncompensated 0.85 electrons. Indeed, this small moment, compared to the about 4 times higher Mn moment is consistent with the strength observed in the Mn and Ni-XRMR. In case of the wide interface, Mn is in a non-FM state, and therefore providing no effective exchange coupling to the Ni-side. On the other hand it is well known, that *Re*NiO_3_ tends to have antiferromagnetic double layer structures^35^. Therefore, Ni at the wide interface, now being FM ordered because of the doping assisted double exchange like FM interactions, couples itself antiferromagnetically to the Ni layer on the other side of each LNO layer. In addition, we would like to mention the influence of Ca doping, where a slight doping of a few percent stabilizes the metallic phase in Sm_1−x_Ca_x_NiO_3_^36^. As this type of Ca doping could be also present here close to the interfaces, this could also increase the FM interaction strength to the FM-LCMO.

## Conclusions

Here we report the XRMR based observation of a spontaneous magnetic depth profile, where different interface electronic structures in the LCMO–LNO superlattices have been observed as a function of growth order. A consistent simple charge model is developed explaining qualitatively our nonmagnetic and magnetic resonant scattering profiles in terms of charge and spin profiles.

At the sharper interface the Mn valence increases over a wider range by about half a valence electron, while for Ni the valence decreases by the same number of electrons, obeying charge compensation^2^. This is accompanied by a ferromagnetic order of the LCMO and LNO interface with an enhanced Mn moment. In contrast, the valence stays fixed at the bulk values for LCMO and LNO at the wider, stronger intermixed interface. Here, the charge transfer cancels the Mn magnetic polarization due to the lack of double exchange coupling. This reduced FM order on one, wide side of the LNO component allows AFM coupling of the Ni ions along inside the LNO layer creating opposite magnetized interfaces with vanishing average Ni moment, which is the usual magnetic configuration on the other RE based Ni oxides. As the STEM results do not reveal a significant structural variation not directly visible superlattice growth asymmetry, as previously observed^33^, is likely to be the initial trigger for these observed strong charge and spin profiles.

## Method part

### Preparations and structural analysis

Sets of [*n-*LNO/*m-*LCMO]_l_ heterostructures and superlattices (SLs) have been grown using a conventional pulsed laser deposition technique (PLD) on 5 × 5 mm^2^ single crystal (001)-oriented SrTiO_3_ (STO) substrates. Where *n, m* is the number of unit-cell (u.c.) of each layer and l is the total number of modulation length. For the PLD process, a KrF-excimer laser with a wavelength of λ = 248 nm was used with the photon fluency adjusted to 1.6 J/cm^2^, and the pulse frequencies to 2 Hz and 1 Hz for LNO and LCMO, respectively. For the deposition, the substrates were heated to 780 °C in Argon with an oxygen partial pressure of 0.4 mbar. All of the heterostructure and SLs are grown at this condition. After the deposition, the films are cooled down to 530 °C with ramping rate of 5 °C/min, while the oxygen partial pressure was increased to 1 bar. The samples were annealed for 30 mints to obtain complete oxygenation.

### High resolution scanning transmission electron microscope (STEM)

High angle annular dark-field (HAADF) and EELS spectrum imaging were performed on a Nion UltraSTEM 100 operated at 100 kV with a 30 mrad convergence semi-angle and a fifth-order aberration corrector. Spectra were acquired with a Gatan PEELS 666 spectrometer retrofitted with an Andor iXon 897 electron-multiplying charge-coupled device (EMCCD) camera. A dispersion of 1.2 eV per channel and an exposure time of 50 ms per spectra were used to record the oxygen K-edge, Mn and Ni L_2,3_ edges and La M_4,5_ edge in a 32 by 128 pixel spatial grid.

### X-ray magnetic reflectivity (XRMR)

The magneto-optical profile was measured using X-ray resonant magnetic reflectivity (XRMR)^26^. The XRMR experiments were performed using a UHV reflectometer ERNSt at the UE56 beamline at Bessy/HZB Berlin^27^. The beamline has a photon energy resolution of about ΔE/E of ~ 10^−4^. The base pressure of the diffractometer chamber was kept lower than 10^−9^ mbar. The samples were aligned with their surface normal in the scattering plane and measured at temperatures of 88 K and 35 K. The measurements were carried out in the specular θ–2θ reflection geometry at several non-resonant photon energies as well as energies at the Mn-L_2,3_ resonance (~ 635–660 eV) and at the Ni-L_2,3_ resonance (~ 850–870 eV).

## Supplementary Information

Supplementary Information.

## Data Availability

The datasets used and analyzed during the current study available from the Correspondence and requests for materials should be addressed to S.S. (soltan@is.mpg.de) and E.G. (goering@is.mpg.de).
