# Supplementary material for: Ferromagnetic order controlled by the magnetic interface of LaNiO3/La2/3Ca1/3MnO3 superlattices
Source: Sci Rep. 2023 Mar 8;13:3847. doi: 10.1038/s41598-023-30814-6 (PMC9995495; doi:10.1038/s41598-023-30814-6)
Supplement: Supplementary file 1 — Supplementary Information. [file 41598_2023_30814_MOESM1_ESM.docx]

**Ferromagnetic order controlled by the magnetic interface of LaNiO_3_/La_2/3_Ca_1/3_MnO_3_ superlattices**

**S. Soltan^1-3^, S. Macke^3^, S. Ilse^2^, T. Pennycook^4,5^, Z. Zhang^6^, G. Christiani^3^, E. Benckiser^3^,** **G. Schütz^2^, E. Goering^2^**

^1^Physics Department, Faculty of Science, Helwan University, 11798-Helwan, Cairo, Egypt

^2^Max-Planck-Institute for Intelligent Systems, Heisenbergstr. 3, D-70569 Stuttgart, Germany

^3^Max-Planck-Institute for Solid State Research, Heisenbergstr. 1, D-70569 Stuttgart, Germany

^4^ EMAT, University of Antwerp Campus Groenenborger, 2020 Antwerp, Belgium

^5^ Faculty of Physics, University of Vienna Boltzmanngasse 5, 1090 Vienna, Austria

^6^ Erich Schmid Institute of Materials Science, Austrian Academy of Sciences, A-8700 Leoben, Jahnstraße 12, Austria

**Supplementals:**

**1- High resolution scanning transmission electron microscopy (STEM):**

High angle annular dark-field (HAADF) and EELS spectrum imaging were performed on a Nion UltraSTEM 100 operated at 100kV with a 30 mrad convergence semi-angle and a fifth-order aberration corrector. Spectra were acquired with a Gatan PEELS 666 spectrometer retrofitted with an Andor iXon 897 electron-multiplying charge-coupled device (EMCCD) camera.

| 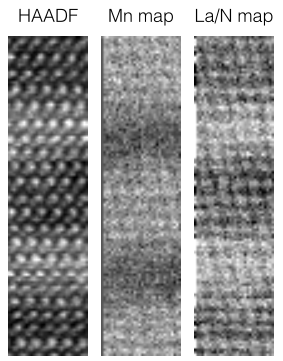 HAADF | La + Ni | Mn |  |
| --- | --- | --- | --- |
| 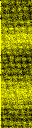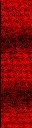 | | | Wide  Narrow  Wide  Narrow |
| Figure S1: Atomic resolution EELS maps showing the Mn–L_2,3_ edge and the combined signal of the overlapping La-M_4,5_ and Ni-L_2,3_ edges adjacent to a simultaneously acquired HAADF image. The maps indicate the distribution of Mn and La and Ni in the different layers and reflect the good epitaxial film quality. | | | |

A dispersion of 1.2 eV per channel and an exposure time of 50 ms per spectra were used to record the oxygen K-edge, Mn and Ni L_2,3_ edges and La M_4,5_ edge in a 32 by 128 pixel spatial grid. Figure S1 shows maps of the Mn and La plus Ni signals alongside the simultaneously recorded HAADF image. The edge onsets Mn L_2,3_ at 640 eV, La M_4,5_ at 832 eV, Ni L_2,3_ at 855 eV. As the La and Ni edges overlap we provide a simple background subtracted map of the entirety of both edges combined. Significant drift is present due to the slow scan rate needed for the spectrum imaging. Nevertheless, the layers can be distinguished and the films can be seen to be of good epitaxy and relatively symmetric.

In order to clarify the interface modulations and the different roughness issue we have analyzed the HAADF image, as shown in Figure S2. The raw data curve (blue) has calculated by horizontal line wise averaging the gray scale image intensities. In the next step, two different smoothing methods were applied to highlight the different material layers, which can be seen in the raw data as a low frequency modulation. As the first smoothing method (green) a local 2^nd^ order polynomial regression with weighted linear least squares were used.

| 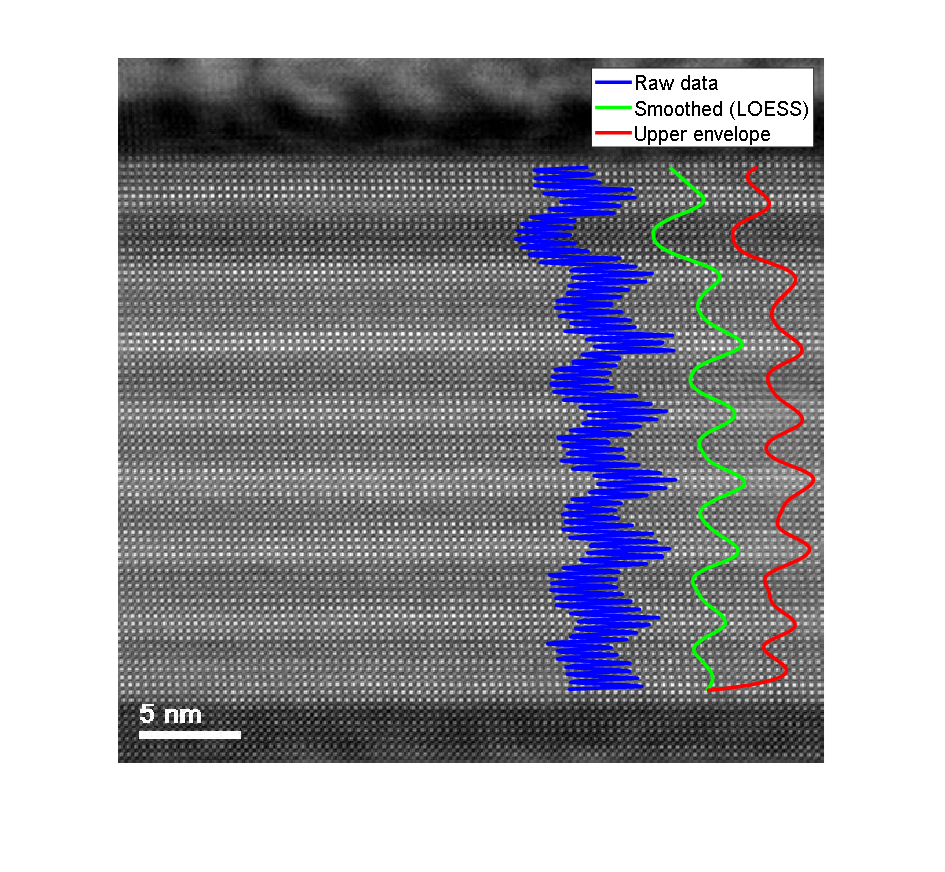 |
| --- |
| Figure S2: The HAADF image of the [3u.c.-LNO/5u.c.LCMO]_8_ superlattices with different fitting methods for the interfaces. The raw data curve (blue) has calculated by line-wise averaging the gray scale image intensities in x-direction. Green and red lines the fitting results of the raw data curve blue. |

The second method (red) consists of an upper maximum peak envelope interpolation, using a spline fit over local maxima. As a result, both fitting methods show very similar behavior and no distinct layer growth profile difference between the upper and lower LCMO interfaces, as suggested by our resonant XRMR fits. This is a clear indication that the obtained XRMR results are not mainly based on asymmetric growth properties, and therefore more likely on spontaneous electronic structure symmetry breaking as discussed in the main text.

**2- XRMR Hysteresis loops:**

In order to monitor the correlation between the Mn and the Ni sublattice magnetizations, we performed element specific hysteresis loops, measured in reflection mode at an angle of 2θ = 29° for both elements. Figure S3a-b shows the resonant reflectivity curves as a function of the angle of incidence. In Fig. S3c-d the corresponding element specific hysteresis loops are presented for two different temperatures, 35 K and 88 K. Both curves are normalized to saturation, and therefore providing the same sign. It is clear that Mn and Ni behave in a strongly coupled way, showing similar low coercive behavior at 88 K and high coercivity at 35 K, with almost the same coercive fields. It should be noted that small details in the shape and related slight deviations between Mn and Ni could not be simply attributed to real differences in switching behavior. This is a well-known phenomenon to XRMR based hysteresis loops and based on nonlinear field dependent variations in the XRMR asymmetry [1]. We also want to mention, that the XRMR based hysteresis loops are normalized to their corresponding maximum values.

**3- Mn XRMR model verification**

In order to clearly demonstrate the validity of our XRMR results we also present other magnetization profiles. In Figure S4 we show various simulated magnetic asymmetry curves in comparison to the measured Mn L_3_ edge XRMR asymmetry. In order to prove the increased magnetic moment on the left side and the reduced moment at the right side of each LCMO layer, our first test Mn Profile (Fig. S4 blue curve) still assumes an asymmetric shape behavior for left and right interfaces, as suggested by the chemical best fit profile (Fig. S4 black curve), but in contrast to the results in the main text, where the magnetization is strongly increased (Fig. S4 black curve) the magnetization has been held to its maximum expected bulk value over the full width of the LCMO layer (Fig. S4 blue curves). So there is no increase on the left and no “dead” like layer on the right, just the chemical profiles are superimposed to the magnetic layer. The resulting asymmetry provides clearly less agreement with the experimental data in the momentum transfer range of 0.17-0.45 1/Å. This is strongly suggesting a significant increased Mn magnetic moment at the so called narrow interface on the left side of the LCMO layer. Here the maximum height has been held fixed and the shape of the Mn profile has been held free to obtain best fit. The resulting shape was almost the same as provided by the free full fit as presented in the main text. This also suggest the presence of an asymmetric profile for left and right sides for each LCMO layer.

|  |
| --- |
| Figure S3: Panel (a) and (b) shows the XRMR at Mn-L3 and Ni-L3 edges. (c) and (d) show the normalized magnetizations for Mn-L3 and Ni-L3 edges at 88K and 35K respectively. |

In addition, we made a magnetic test profile No. 2, which was forced to have a symmetric behavior for left and right interfaces. The best fit result is also presented (Figure S4 green curve). As a result of a symmetric profile the asymmetry is now in much less agreement with respect to the experimental data. In the momentum transfer range of 0.17-0.45 1/Å the profile does not show a clear long period substructure with the sign change at about 0.35 1/Å anymore. Here also short wavelength oscillations are almost damped out. In addition, the agreement in the lower momentum transfer range up to 0.17 1/Å is clearly less.

| 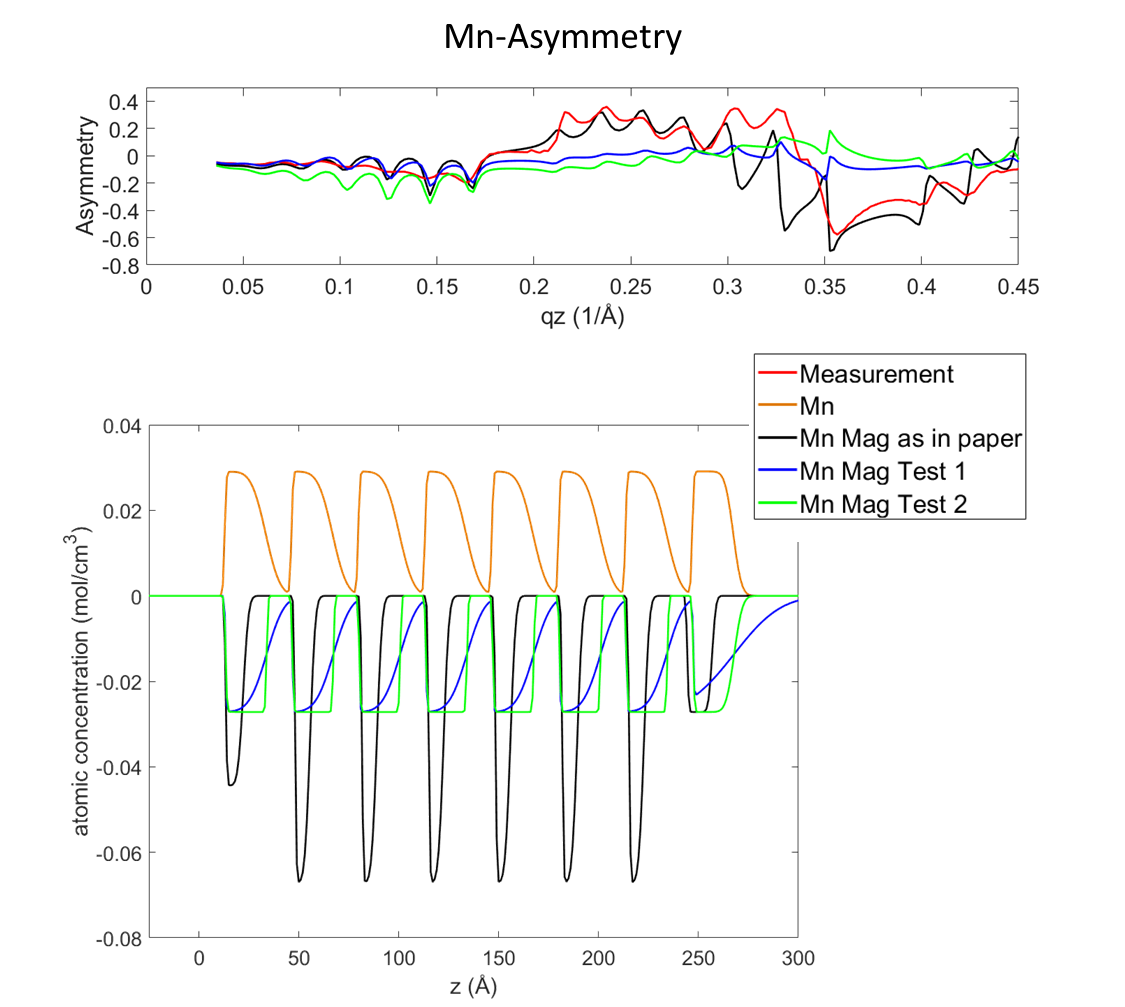 |
| --- |
| Figure S4: Upper part: Mn magnetic XRMR asymmetry related to different magnetization profiles together with the Mn density profile. Lower part: Corresponding Mn magnetization profiles. Compare to Mn profiles in the main text. |

This comparison clearly demonstrates the validity of our best fit Mn profile as presented in the main text, with a strong increased magnetic moment at the left interface and a dead layer like reduction at the right interface.

**4- Ni XRMR model verification**

Similar to the previous comparison we now present the magnetic XRMR asymmetries for the Ni L_3_ and L_2_ edges. Here the magnetic profiles have been held symmetric with respect to the LNO profile. In the upper part of Figure S5 the best fit Ni magnetization profile for the L_3_ edge is shown. It has inverse magnetization orientation at the interfaces (positive sign) and a slightly parallel orientation at the center (negative sign). Even for this best fit, the resulting asymmetry is far from the experimental curvature (Figure S5 upper right part).

In the lower part of Figure S5 the best fit Ni magnetization profile for the L_2_ edge is shown. Here the Ni magnetization is always parallel with respect to the Mn magnetization. Similar to the above presented L_3_ edge results, the resulting best fit asymmetry is far from the experimental curvature (Figure S5 lower right part). In contrast the up-down magnetization Ni profile as presented in the main part in Figure 5 provides a simulated asymmetry, which is in almost perfect agreement with the measurement. Here both best fit results are far away from the measurement and also very different with respect to each other, while the best fit using antiparallel oriented Ni magnetizations are almost the same for the L_2_ and L_3_ edges, even for free fit profiles. This is a very clear confirmation that the obtained profile is a good representation of the real profile present in the sample.

| 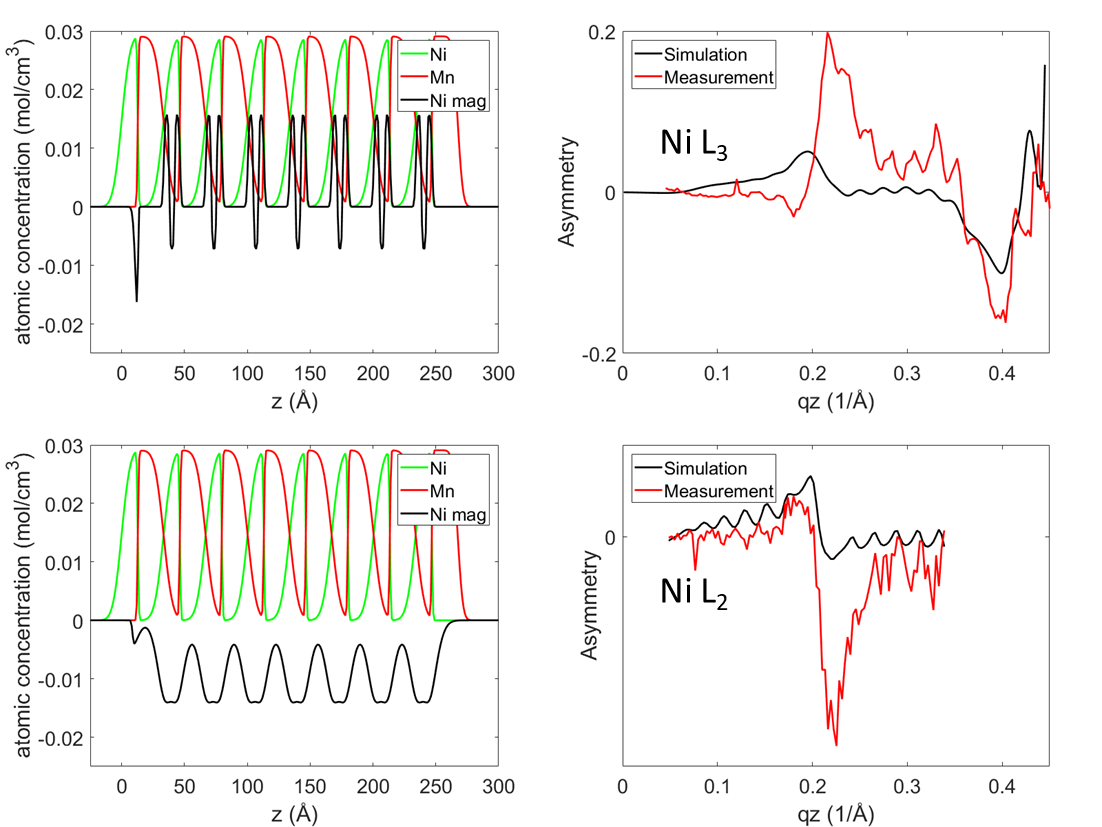 |
| --- |
| Figure S5: Left: Chemical Mn (red) and Ni (green) profiles are shown together with the best fit for forced symmetric Ni magnetization profiles (black curve) for the Ni L_3_ edge (above) and L_2_ edge (below). Right: Corresponding XRMR asymmetry simulation results in comparison with the measured XRMR asymmetry. |

**(4) First-order reversal curves (FORC):**

In these studies, we have used the nano-magnetic Kerr effect (Nano-MOKE) of [3u.c.-LNO/5u.c.LCMO]_8_ for the mapping of exchange-bias and spectroscopic. Figure S6 shows the first-order reversal curves (FORC) diagram exhibits two distinct peaks. A high coercivity peak H_c_ = 450 Oe/H_u_ and a lower coercivity peak that is broadened towards higher coercive fields and interaction peaks. The narrow distribution of the high coercivity peak, both in H_c_ and H_u_ direction, indicates that it results from a distinct magnetic switching event that is not subject to any interaction fields except the externally applied field [2]. Considering the element specific major loop hysteresis (XMCD), this is likely a Mn species that is responsible for the high field widening of the major loop. This also agrees with the small peak intensity, as

| 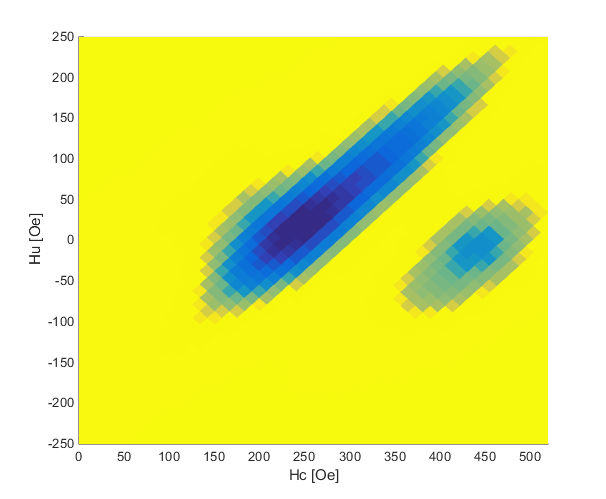 |
| --- |
| Figure S6: the first-order reversal curves (FORC) diagram of the interaction-field (H_u_) versus coercivity-field (H_c_) at 25K. |

this switching event only results in a minor total change in magnetization.

The low coercivity peak in the FORC diagram, however, is more intriguing as it exhibits both a distribution and an interaction field signature. This is likely due to Mn interacting with Ni at the LCMO/LNO interfaces. This is also in agreement with the element specific hysteresis loops as the low field switching event occurs both for Mn and Ni, which indicates that this is a coupled process. This assignment is both in agreement with a distribution in switching field (H_sw_ = H_c_ + H_u_) and the presence of an interaction field. The shape of the element specific major loop already gives a hint at a distribution of switching fields that is found in the FORC diagram [2-3]. More intriguing, however, is the distribution of interaction field that indicates a rather strong interaction between Mn and, likely, Ni. The effective interaction field that is exerted on the Mn amounts to almost 200 Oe and, thus, the same order of magnitude as the intrinsic coercivity. As the interpretation as Preisach distribution, i.e. the direct interpretation as coercivity and local interaction, breaks down for the case of coercivity and interaction on the same order of magnitude the absolute quantification of the interaction field is not straightforward [2-3]. However, it can be concluded that the microscopic interaction of Mn and Ni is on the same order of magnitude as the origin of the intrinsic coercivity, i.e. it can be assumed that the interaction is on the order of exchange interaction.

(5) **X-ray magnetic circular dichroism (XMCD)** :

Here we also show the XMCD spectra, which are also necessary to provide the optical properties needed for XRMR fitting, see Figure S7(a-b). A clear ferromagnetic response is visible for the Mn in LCMO, which is smaller than for bulk LCMO, as expected from our reduced top layer magnetization and the other magnetization reductions in the Mn XRMR magnetization profile. In the case of LNO the XMCD is very small. As the topmost LNO layer Ni is ferromagnetically coupled to the Mn we indeed expect some FM signal, as the total electron yield used here is strongly damped for deeper layers, a very small XMCD remains. As already mentioned in the main text, we did not use the original Ni L2,3 edge XMCD from our sample, as the Ni L3 part is superimposed onto the large La M4,5 edges.

|  |
| --- |
| Figure S7:(a) Normalized X-ray absorption spectroscopy XAS and X-ray magnetic circular dichroism (left and right) for the Mn-L3 edge at 87K, and (b) for Ni-L3 edge, where the very small Ni-XMCD signal has been enhanced by a factor of 20 for better visibility. |

**(6) Transport and magneto-resistance of the [3 u.c. LNO- 5 u.c. LCMO]_8_ superlattices:**

Figure S8(a-b) shows that the superlattices are metallic in all temperature ranges. Furthermore, the resistance versus magnetic field (MR) shown in Figure S8b for two different magnetic orientations parallel (blue) and perpendicular (red) to the film plane at temperatures from 5K to 200K also shows a negative magneto-resistance, which is typical behavior of metallic states.

|  |
| --- |
| Fig. S8: (a) resistance versus temperature for the [3 u.c. LNO- 5 u.c. LCMO]_8_ superlattices which shows a metallic behavior in all temperature range except of up-turn resistance below 25K. |

**References:**

[1] S. Macke & E. Goering, Journal of Physics: Condensed Matter, **26** (2014) 363201.

[2] J. Gräfe et al., Phys. Rev. B 93 (2016) 104421.

[3] F. Groß et al., Phys. Rev. B 93 (2019) 104421.
